# Lag-time effects of vaccination on SARS-CoV-2 dynamics in German hospitals and intensive-care units

**DOI:** 10.3389/fpubh.2023.1085991

**Published:** 2023-04-11

**Authors:** Bruno Enagnon Lokonon, Yvette Montcho, Paul Klingler, Chénangnon Frédéric Tovissodé, Romain Glèlè Kakaï, Martin Wolkewitz

**Affiliations:** ^1^Laboratoire de Biomathématiques et d'Estimations Forestières, Université d'Abomey-Calavi, Cotonou, Benin; ^2^Faculty of Medicine and Medical Center, Institute of Medical Biometry and Statistics, University of Freiburg, Freiburg im Breisgau, Germany

**Keywords:** delayed effects, vaccination, non-pharmaceutical interventions (NPIs), linear lag models, COVID-19, policy decisions

## Abstract

**Background:**

The Efficacy and effectiveness of vaccination against SARS-CoV-2 have clearly been shown by randomized trials and observational studies. Despite these successes on the individual level, vaccination of the population is essential to relieving hospitals and intensive care units. In this context, understanding the effects of vaccination and its lag-time on the population-level dynamics becomes necessary to adapt the vaccination campaigns and prepare for future pandemics.

**Methods:**

This work applied a quasi-Poisson regression with a distributed lag linear model on German data from a scientific data platform to quantify the effects of vaccination and its lag times on the number of hospital and intensive care patients, adjusting for the influences of non-pharmaceutical interventions and their time trends. We separately evaluated the effects of the first, second and third doses administered in Germany.

**Results:**

The results revealed a decrease in the number of hospital and intensive care patients for high vaccine coverage. The vaccination provides a significant protective effect when at least approximately 40% of people are vaccinated, whatever the dose considered. We also found a time-delayed effect of the vaccination. Indeed, the effect on the number of hospital patients is immediate for the first and second doses while for the third dose about 15 days are necessary to have a strong protective effect. Concerning the effect on the number of intensive care patients, a significant protective response was obtained after a lag time of about 15–20 days for the three doses. However, complex time trends, e.g. due to new variants, which are independent of vaccination make the detection of these findings challenging.

**Conclusion:**

Our results provide additional information about the protective effects of vaccines against SARS-CoV-2; they are in line with previous findings and complement the individual-level evidence of clinical trials. Findings from this work could help public health authorities efficiently direct their actions against SARS-CoV-2 and be well-prepared for future pandemics.

## 1. Introduction

The severe acute respiratory syndrome coronavirus 2 (SARS-CoV-2) that emerged in China in late 2019 has caused major public health concerns and continues to spread worldwide (1–3). There have been a total of 614 million confirmed cases globally, with over 6 million deaths reported, as of August 31, 2022, WHO (4). In Germany, the first COVID-19 case was reported on January 27, 2020, in Bavaria and by March 1, 2020, more than 100 cases were reported (5). Non-Pharmaceutical Interventions (NPIs) have quickly been promoted by the federal government including schools, kindergartens, universities, borders for travelers closing as well as national curfew and contact ban (6). The infection rate decreased following these measures, however, in mid-July 2020, the number of cases started to rise again due to relaxation (7). NPIs have been sufficiently effective in curtailing and mitigating the burden of the pandemic during at least its first waves (6, 7), however, some of them such as containment and travel ban could not be maintained for long times. It was then believed that the use of vaccines combined with some control measures may be necessary to effectively curtail and eliminate COVID-19 (8).

In Germany, the vaccination program began on December 27, 2020, and as of August 31, 2022, 77.66% of all German population have been fully vaccinated (9). The most used vaccines in Germany are BioNTech (95% of efficacy), Moderna (94.1%), AstraZeneca (67%), and Johnson & Johnson (67%) (10–12).

The effect of vaccines is manifold as they act on the individual as well as the population level (13). On the individual level, vaccines aim to reduce the risk of acquiring the infection and transmission but also the clinical consequences once infected. The gold standard study designs to assess vaccine efficacy are randomized placebo-controlled trials. In addition, cohort and case-control studies are used to measure the vaccine effectiveness during real-world conditions (13). For the SARS-CoV-2 pandemic, several studies have shown vaccines efficacy and effectiveness (14–17).

Contrary to cohort and case-control studies, ecological or trend studies compare results on population level over time with varying vaccine coverage (13). The classical approach in ecological studies is to extrapolate from time trends before vaccine introduction, thus creating counterfactual settings which are essential for causal inference. However, the variants of SARS-CoV-2 and their different impact on the pandemic dynamic have made extrapolation extremely difficult.

Moreover, several mathematical models have been developed to predict and assess the impact of vaccination on the transmission dynamics of COVID-19. Gnanvi et al. (18) performed a systematic and critical review on the reliability of predictions of the modeling techniques on COVID-19 dynamics. Dashtbali and Mirzaie (19) used a Susceptible, Exposed, Infected, Hospitalized, Recovered, and Death compartmental model and found that, in the German population, the number of infected cases at the epidemic peaks decreases by increasing the vaccine coverage. Wollschläger et al. (20) applied a multivariable logistic regression on data from the German federal state of Rhineland-Palatinate and concluded that vaccination coverage was associated both with a reduction in the age-groups proportion of COVID-19 fatalities and of reported SARS-CoV-2 infections. Braun et al. (21) developed an effect model based on the Batman-SIZ algorithm for modeling the effect of vaccination on the course of the pandemic in Germany. They obtained that, the effect of vaccination in reducing the daily number of new infections, the total number of infections and the occupancy of intensive-care facilities in hospitals is proportional to the speed with which the target population are vaccinated. Springer et al. (22) used linear regression on the 4th corona wave in Germany and showed that there is a negative correlation between the vaccination rate and the infection incidence. Campos et al. (23) used a membrane computing model for simulating the efficacy of vaccines on the epidemiological dynamics of SARS-CoV-2. They obtained that generalized vaccination of the entire population (all ages) added little benefit to overall mortality rates. However, elderly-only vaccination, even without general interventions directed to reduce population transmission, is sufficient for dramatically reducing mortality. Stiegelmeier et al. (24) proposed a p-fuzzy system in order to model the COVID-19 epidemic evolution under the effect of vaccination in Brazil. They concluded that the level of infestation tends to decrease as the number of people vaccinated increases. Sepulveda et al. (25) constructed a mathematical model based on a nonlinear system of delayed differential equations to investigate the qualitative behavior of the COVID-19 pandemic under an initial vaccination program. They found that if the basic reproduction number is less than one and the time delays are less than some critical threshold, then the disease-free equilibrium is locally stable. Thus, if public health authorities are able to reduce transmission rates and increase vaccination rates, the burden of the COVID-19 pandemic can be reduced.

Despite the contributions of these studies, they showed some limitations. First, the classic mathematical models of epidemiological prediction are quite useful, but deterministic, demonstrating only the average behavior of the epidemic, which makes it difficult to quantify uncertainty (26). Second, the effect of vaccination on COVID-19 reported data may not be linear. Third, vaccination may also show effects that are delayed in time, requiring assessment of the temporal dimension of the exposure-response relationship (27). In addition, the previous studies ignore the seasonal patterns of COVID-19 and the long-term trends in the data. Indeed, the main challenge of modeling the effects of exposure like vaccination on COVID-19 reported data lies in the additional temporal dimension needed to express this relation, as the effects depend on both intensity and timing of past exposure (28). Although several studies have assessed the effects of vaccination on COVID-19 dynamics, very few have considered its delayed effects.

The aim of this paper is to use an ecological or trend study to evaluate the way how vaccine coverage of the German population is associated with the number of SARS-CoV-2 patients in general hospitals as well as intensive care units. Instead of extrapolating from time trends before vaccine introduction, we adjusted for the remaining time trends by natural splines with a high degree of freedom. We applied a flexible modeling framework by Gasparrini et al. (29) that can simultaneously represent exposure-response dependencies and delayed effects. This family of models is called distributed lag linear models (DLMs). Specifically, we evaluated the effects of vaccination on the number of prevalent hospital patients (hospital cases) and intensive care unit patients (ICU cases) through three separate analyzes by considering people vaccinated with one dose (i), people vaccinated with two doses (ii), and people vaccinated with three doses (iii). We focused on these two outcomes (hospital cases and ICU cases) since for the COVID-19 pandemic, controlling hospital and ICU admissions was for German public health authorities, an important factor in saving the lives of the patients (30).

## 2. Methods

### 2.1. Model framework

#### 2.1.1. General form

To describe the time series of outcomes *Y*_*t*_, the general form of the model is Gasparrini et al. (29):


(1) $$\begin{array}{l} f\left(E\left(Y_{t}\right)\right)=\alpha +\overset{J}{\underset{j=1}{\sum }}s_{j}\left(x_{tj};\beta_{j}\right)+\overset{K}{\underset{k=1}{\sum }}\gamma_{k}u_{tk}, \end{array}$$

where *f* is a monotonic link function and *Y*_*t*_ is a count time series response variable, with *t* = 1, …, *n*, following a distribution that belongs to the exponential family. *s*_*j*_ defines a smoothed relationship between *x*_*j*_ and *Y*_*t*_ through a coefficient β_*j*_. *u*_*k*_ represent confounding variables and γ_*k*_ the related coefficients.

In this work, the outcomes *Y*_*t*_ are daily numbers of prevalent hospital patients and intensive care unit patients. According to Cameron and Trivedi test (31), these outcomes are overdispersed (α = 2903.716, *p* < 0.0001 for hospital cases and α = 1201.386, *p* < 0.0001 for ICU cases). We therefore considered a quasi-Poisson model with E(*Y*) = μ; V(*Y*) = ϕμ, and a canonical log-link in Equation (1). Our motivation to choose the quasi-Poisson model (instead of other alternatives such as the Negative Binomial model) falls in the straightforward interpretation of the results.

#### 2.1.2. Basic functions and delayed effects

The definition of the basis functions relies on two steps. In the first step, the relationship between *x*_*j*_ and *f*(*E*(*Y*_*t*_)) is represented by *s*(*x*), and is set in Equation (1) as a sum of linear terms (29). This relationship is carried out by the choice of a basis, a space of functions of which *s* is an element (32). The associated basis functions are some known transformations of the original variable *x* that generate a new set of variables, termed basis variables (29). Several basis functions have been proposed, and common functions assuming smooth curves, like polynomials or spline functions (33, 34). The basis function is expressed as follows:


(2) $$\begin{array}{l} s\left(x_{t};\beta \right)={\text{z}}_{t}^{⊤}\cdot \beta , \end{array}$$

where $z_{t}^{⊤}$ is the *t*th row of the *n*×*v*_*x*_ basis matrix *Z*. The basis dimension *v*_*x*_ equals the degrees of freedom (*df*) spent to define the relationship in this space. The unknown parameters are estimated including *Z* in the design matrix of the model in Equation (1).

In the second step, the delayed effects are considered as an additional dimension. The outcome at a given time *t* is then explained in terms of past exposures *x*_*t*−*l*_, where *l* (the *lag*) represents the elapsed period between exposure and response, here between vaccination and response.

In this study, the maximum lag is fixed at *L* = 30 days, based on previous estimates of the incubation period for COVID-19 (35, 36).

#### 2.1.3. The distributed lag linear models

For a maximum lag *L*, the additional lag dimension can be expressed by the *n*×(*L*+1) matrix **Q**, such as:


(3) $$\begin{array}{l} {\text{q}}_{t.}={\left[x_{t},\ldots ,x_{t-\ell },\ldots ,x_{t-L}\right]}^{⊤}, \end{array}$$

with **q**_*t*_. as the *t*th row of **Q**. The vector of lags ℓ = [0, …, ℓ, …, *L*]^⊤^ corresponds to the scale of the additional dimension. DLMs are specified by the definition of a cross-basis, a bi-dimensional functional space describing at the same time, the shape of the relationship along the predictor *x* and its distributed lag effects (37). DLMs apply simultaneously the two transformations described in Equations (2), (3). A DLM is expressed by Gasparrini et al. (29):


(4) $$\begin{array}{l} s\left(x_{t};\eta \right)=\overset{v_{x}}{\underset{j=1}{\sum }}\overset{v_{\ell }}{\underset{k=1}{\sum }}{\text{r}}_{tj}^{⊤}{\text{c}}_{\cdot k}\eta_{jk}={\text{w}}_{t}^{⊤}\eta , \end{array}$$

where **r**_*tj*_ is the vector of lagged exposures for the time *t* transformed through the basis function *j*, **C** is an (*L* + 1) × *v*_ℓ_ matrix of basis variables derived from the application of the specific basis functions to the lag vector ***ℓ***, the vector **w**_*t*_ is obtained by applying the *v*_*x*_·*v*_ℓ_ cross-basis functions to *x*_*t*_ and ***η*** a vector of unknown parameters.

### 2.2. The data

**Figure 1:**
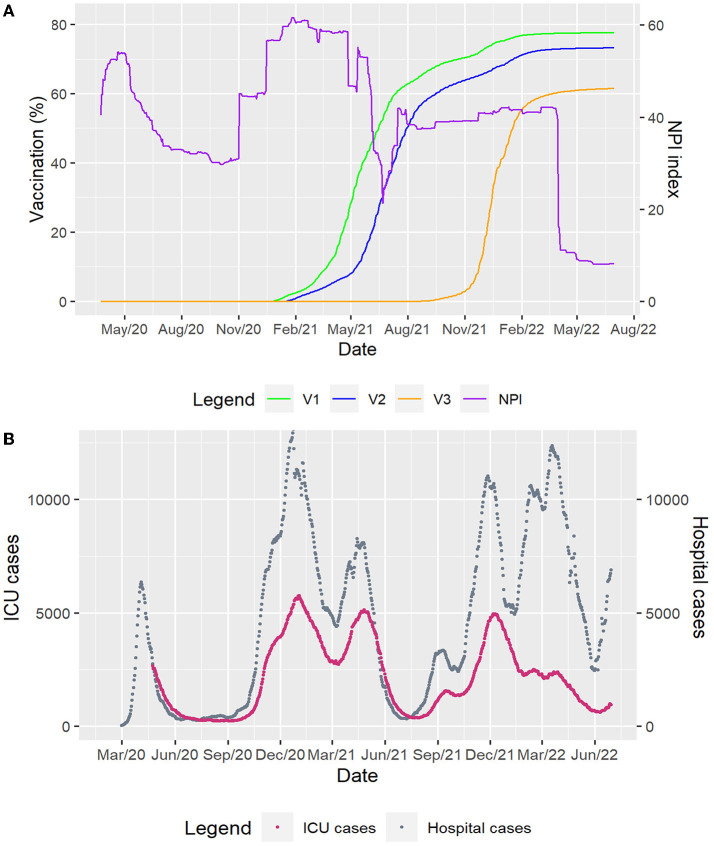
Time series of German data considered. **(A)** The predictors represented by the daily cumulative proportions of people vaccinated with one dose (*V*_1_), two doses (*V*_2_), three doses (*V*_3_) and the non-pharmaceutical interventions (*NPI*) index. **(B)** The outcomes: the daily numbers of prevalent hospital patients (hospital cases) and intensive care unit patients (ICU cases).

The time series of the COVID-19 data were extracted from the Robert-Koch-Institute website (https://www.rki.de/) and www.corona-datenplattform.de, data platforms for scientific research. The predictors were the daily cumulative proportions of people vaccinated with one dose (*V*_1_), two doses (*V*_2_), three doses (*V*_3_) and the non-pharmaceutical interventions (*NPI*) index (Figure 1A). The outcomes were the number of prevalent hospital patients (hospital cases) and intensive care unit patients (ICU cases) (Figure 1B). Hospital cases were collected from March 1, 2020, to June 30, 2022, while ICU cases were collected from March 24, 2020, to June 30, 2022.

### 2.3. Application of DLM to German data

We were interested in the effects of the daily cumulative proportion of people vaccinated with one dose (*V*_1_), two doses (*V*_2_) and three doses (*V*_3_), respectively. As *V*_1_, *V*_2_ and *V*_3_ are highly correlated, we first regressed each series of proportions against the two others and collected the residuals (38), which were considered as a second variable in the DLM. We performed three preliminary separate analyzes as follows:

For the effect of *V*_1_, we considered:


(5) $$\begin{array}{l} model_{1}:log\left(V_{1}\right)\sim log\left(V_{2}\right)+log\left(V_{3}\right)+\epsilon_{a}. \end{array}$$

For the effect of *V*_2_, we used:


(6) $$\begin{array}{l} model_{2}:log\left(V_{2}\right)\sim log\left(V_{1}\right)+log\left(V_{3}\right)+\epsilon_{b}. \end{array}$$

For the effect of *V*_3_, we applied:


(7) $$\begin{array}{l} model_{3}:log\left(V_{3}\right)\sim log\left(V_{1}\right)+log\left(V_{2}\right)+\epsilon_{c}. \end{array}$$

The DLMs considered to assess the effects of *V*_1_, *V*_2_, and *V*_3_ on the hospital and ICU cases were defined, respectively, as follows:


(8) $$\begin{array}{l} \log\left(E\left(Y_{t}\right)\right)=\alpha_{1}+ns\left(time,df\right)+\beta_{1}V_{1,t}+\gamma_{1}\epsilon_{a,t}+\lambda_{1}NPI_{t}, \end{array}$$

(9) $$\begin{array}{l} \log\left(E\left(Y_{t}\right)\right)=\alpha_{2}+ns\left(time,df\right)+\beta_{2}V_{2,t}+\gamma_{2}\epsilon_{b,t}+\lambda_{2}NPI_{t}, \end{array}$$

(10) $$\begin{array}{l} \log\left(E\left(Y_{t}\right)\right)=\alpha_{3}+ns\left(time,df\right)+\beta_{3}V_{3,t}+\gamma_{3}\epsilon_{c,t}+\lambda_{3}NPI_{t}, \end{array}$$

where *Y*_*t*_ represents the hospital or ICU cases, log, the natural log function, α_1_, α_2_, α_3_ are the models intercepts, ϵ_*a*_, ϵ_*b*_, and ϵ_*c*_ are the residuals extracted from Equations (5)–(7). The variable *time* was set in the model to consider long-term trends and to account for some of the pandemic patterns, such as variants and seasonal variations, which are not explained by remaining predictors. In Equations (8)–(10), *V*_1, *t*_, *V*_2, *t*_ and *V*_3, *t*_ are the cross-basis functions of the three vaccination doses while ϵ_*a, t*_, ϵ_*b, t*_ and ϵ_*c, t*_ represent the cross-basis functions for the residuals and *NPI*_*t*_, the cross-basis function of the non-pharmaceutical interventions index, considered as confounding variable in the models. The unknown coefficients in the three models are β_1_, β_2_, β_3_, γ_1_, γ_2_, γ_3_, λ_1_, λ_2_ and λ_3_. Moreover, in the Equations (8), (9), and (10), the terms *V*_1, *t*_, *V*_2, *t*_, *V*_3, *t*_, ϵ_*a, t*_, ϵ_*b, t*_, ϵ_*c, t*_ and *NPI*_*t*_ are lagged with lags ℓ∈[0, *L*], where *L* = 30 days represent the maximum lag period. This value was allocated to the maximum lag period considering previous estimates of the incubation period for COVID-19 (35, 36).

The natural cubic spline *ns*, a flexible and effective technique for adjustment for nonlinear confounding effects (39), was used to adjust for the predictors with two degrees of freedom. This number of degrees of freedom was selected after a sensitivity analysis (40). The natural cubic spline *ns* was also used for the variable *time* and the degrees of freedom (dfs) to find the best modeling of the time trend was chosen by minimizing the Quasi-Akaike information criterion (QAIC) (37) as we considered a quasi-Poisson model framework.

The relative count change (RCC) with a 95% confidence interval (*CI*), calculated as a relative increase/decrease in counts of hospital and ICU patients, was used to assess the effects. RCC = 1 means that there is no connection between vaccination and the disease while RCC < 1 and RCC > 1 are related to the reduction and increase in counts of hospital and ICU patients, respectively (41). Contour plots that depend on lag times and values of *V*_1_, *V*_2_, and *V*_3_ were used to visualize the effects. All analyzes were performed in R version 4.2.1 with the package dlnm (37).

## 3. Results

To assess the effects of *V*_1_, the best fitting was obtained for 15 and 16 degrees of freedom for ICU and hospital cases, respectively. Concerning *V*_2_, 16 and 20 degrees of freedom for ICU and hospital cases gave the best fitting. Regarding *V*_3_, 16 and 23 degrees of freedom for ICU and hospital cases showed the best fitting. Supplementary Figures S1, S2 show QAIC values and models fitting (observed data and fitted models).

**Figure 2:**
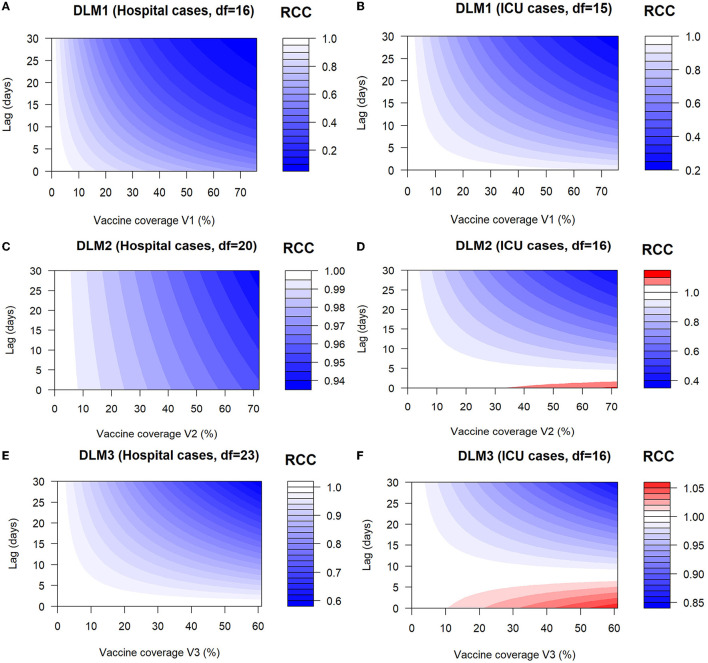
Contour plots of the combined effects of lag times and vaccinations on the relative count change (RCC) of hospital and ICU cases. **(A)** Contour plot of RCC of hospital cases as a function of *V*_1_ and lag times. **(B)** Contour plot of RCC of ICU cases as a function of *V*_1_ and lag times. **(C)** Contour plot of RCC of hospital cases as a function of *V*_2_ and lag times. **(D)** Contour plot of RCC of ICU cases as a function of *V*_2_ and lag times. **(E)** Contour plot of RCC of hospital cases as a function of *V*_3_ and lag times. **(F)** Contour plot of RCC of ICU cases as a function of *V*_3_ and lag times. DLM1, DLM2, and DLM3 represent the distributed lag models for the first, second and third doses.

Figure 2 presents the contour plots of the combined effects of lag times and vaccinations on the relative count change (RCC) of hospital and ICU cases. Overall, low vaccine coverage for the first, second and third doses (0–10%) and short (0–4 days) lag times show no connection between vaccination and the number of patients in hospital and ICU (RCC≈1). However, higher vaccine coverages and longer lag times were associated with a bigger decrease in the number of patients in hospitals and ICUs (RCC < 1). The number of COVID-19 patients in hospitals or ICUs significantly decreases as the vaccine coverage increases. Moreover, there were delayed effects of vaccination according to the doses. Strong protective effects were obtained for a lag time of about 15–20 days after vaccination, when at least about 40% of people are vaccinated.

### 3.1. The effects of *V*_1_ on hospital and ICU cases

Figures 2A, B show the contour plots of the effects of *V*_1_ and lag times on the relative count change (RCC) of hospital and ICU cases. There was no significant effect on hospital and ICU cases (RCC≈1) for low vaccine coverage (0–10%) and short (0–4 days) lag times. Protective effects (decrease in the counts of patients in hospitals and ICUs) were observed around *V*_1_ = 20% with a lag of 5 days, where RCC = 0.80 (95% CI 0.74–0.85) for hospital cases and RCC = 0.92 (95% CI 0.88–0.97) for ICU cases. The number of patients in hospital and ICU (RCC < 1) decreases then sharply as the lag days and vaccine coverage increase. The number of COVID-19 patients in hospitals or ICU significantly decreases (strongest positive effects) for the highest vaccine coverage (77%) and longest lag times (30 days) with RCC = 0.07 (95% CI 0.06–0.09) for hospital cases and RCC = 0.24 (95% CI 0.21–0.27) for ICU cases. Moreover, comparatively, the effects of *V*_1_ on hospital cases are more immediate and intense than on ICU cases.

### 3.2. The effects of *V*_2_ on hospital and ICU cases

Figures 2C, D show the relative count change (RCC) of hospital and ICU cases as a function of *V*_2_ and lag times. Examining the contour plots, no significant effect of *V*_2_ was observed on hospital and ICU cases (RCC≈1) for low vaccine coverage (0–10%). Considering the hospital cases, moderate and immediate positive effects (decrease in counts of hospital patients) were obtained for moderate vaccine coverage *V*_2_ (20–50%) while strong and immediate positive effects were observed for high vaccine coverage *V*_2_ (50–73%). For ICU cases, there were adverse effects (increase in counts of ICU patients) for the highest vaccine coverage (70%) and short (0–3 days) lag times with RCC=1.04 (95% CI 0.79–1.35). From a lag of 5 days, the highest vaccine coverages *V*_2_ were associated with the lowest RCC values, showing strong and positive effects on ICU cases, which last up to 30 days. The effects of *V*_2_ on hospital cases are more immediate and intense than on ICU cases.

### 3.3. The effects of *V*_3_ on hospital and ICU cases

Figures 2E, F show the relative count change (RCC) of hospital and ICU cases as a function of *V*_3_ and lag times. No significant effect of *V*_3_ was noticed on hospital and ICU cases (RCC≈1) for low vaccine coverage (0–10%). Regarding the hospital cases, moderate and immediate positive effects (decrease in counts of hospital patients) were obtained for moderate vaccine coverage *V*_3_ (20–35%) while strong and immediate positive effects were observed for high vaccine coverage *V*_3_ (40–61.50%). For ICU cases, there were adverse effects (increase in counts of ICU patients) for the highest vaccine coverage (60%) and short (0–7 days) lag times with RCC = 1.03 (95% CI 1.00–1.07). From a lag of 10 days, the highest vaccine coverages *V*_3_ were associated with the lowest RCC values and strong and positive effects on ICU cases were observed until a lag of 30 days.

Comparison between vaccine coverages shows that *V*_1_ has a more immediate and intense effect than *V*_2_ and that *V*_2_ also has a more immediate and intense effect than *V*_3_. These observations were made for both hospital and ICU cases. Supplementary Figures S3, S4 present RCC point estimates and their confidence intervals for vaccination coverages V1, V2, and V3 in the cases of hospital and ICU patients, respectively.

## 4. Discussion

In this study, we used an ecological or trend study to assess the effects of vaccination and its lag-time on the number of COVID-19 hospitals and ICU patients in Germany. From our results, there was no significant link between the vaccination coverages *V*_1_, *V*_2_, and *V*_3_ and the number of patients in hospital and ICU for low vaccine coverages (0–10%) and short lag times as the relative count change (RCC) was about 1. This means that regardless of the dose of vaccination received, at least 10% of the population must be vaccinated to expect a beginning protective effect against hospital and ICU admissions. The protective effect is low from 10% and then increases as the vaccination rate increases. As expected, this result supports the point that a high vaccination rate is correlated with a lower number of patients in hospital and ICU (20). We also found that, in the context of Germany, the vaccination takes its strong protective effect when at least approximately 40% of people are vaccinated. This result is consistent with those in Springer et al. (22), which show a negative correlation between incidence and vaccination rate in Germany during the 4th wave where the vaccination rate is above 40%. Our findings are also in line with previous studies showing that COVID-19 vaccines are effective against severe forms of the disease (20, 42).

Furthermore, our results showed a delayed effect of the vaccination according to the doses and outcomes. Indeed, for the hospital cases, the effect is immediate for the first and second doses while for the third dose, a strong protective effect is obtained about 15 days after vaccination. Concerning ICUs cases, there was a lag time of about 15–20 days to obtain a strong protective effect after the first, second and third doses, respectively. These lag times are short compared to that obtained by Li et al. (27) who argued that the lag time for a response to vaccination was at least 40 days. However, contrary to our study, they used the daily reported cases and effective reproduction number as outcomes.

One strength of this study is that our outcomes (hospital and ICU cases) are very specific with low random noise in contrast to other outcomes (general SARS-CoV-2 cases, death cases associated with SARS-CoV-2). German hospitals and intensive care units are legally obligated to report these SARS-CoV-2 data diagnosed with PCR. In Germany, there were about 16.69% and 33.36% deaths among patients admitted to hospital and ICU, respectively (43). These death rates are very high compared to those in the whole population, which is 4.35% (44). It was then important to quantify the effects of vaccination to analyze its contribution to the control of hospital and ICU admissions since public health authorities were most concerned about the scenario where the demand exceeds the capacity of healthcare services (30). To our knowledge, this study is the first that analyzes the effects of vaccination and its lag times on the number of COVID-19 patients in hospitals and ICUs in Germany taking into account long time trends. The findings of this work are relevant and can be applied in other settings and localities. We also included NPIs in our models as a confounding variable since they were maintained at a certain level in the German population while vaccines are distributed. The use of COVID-19 vaccines in combination with the implementation of NPIs is seen as the best alternative to rapidly control the pandemic (45). There is also evidence that an epidemic is likely to rebound immediately after the implementation of a vaccination program if NPIs are completely abandoned (27).

One limitation of our study is that our results are highly dependent on the way we adjusted for the time trends. Moreover, we do not extrapolate hospital cases from time trends before vaccine introduction since emerging variants highly differ from previous variants in terms of transmission, medical condition and burden of disease.

Several observational study designs are discussed in the literature to evaluate the impacts of interventions during an epidemic (13, 46). Digitale et al. (46) reformulated observational studies as pragmatic designs. For these authors, instead of asking retrospective questions about interventions that occurred in the past, the goal should be to prospectively collect data about planned interventions in the future. However, pragmatic designs require more initial planning, community engagement and regulations for human research protection (47). In this study, our design and analysis differ from pragmatic designs. Moreover, we are not interested in estimating vaccine efficacy through randomized trials of individuals or the evaluation of vaccine effectiveness through observational cohort and case-control studies (13). Instead, we aimed to assess the effects of changing vaccine coverage on the number of patients in hospitals and ICUs at a population level. We evaluated the way how vaccination is associated with a decrease in the number of patients in hospitals and ICUs. According to Lipsitch et al. (13), an important consideration of time-trend vaccine effectiveness studies is that the disease outcome under study must be sufficiently specific so that the vaccine's impact on it is likely to be measurable. This was the case for the outcomes considered in this study. Health authorities should therefore consider these results when designing vaccination programs for future pandemics. Knowing the lag times of the vaccination would allow the public health authorities to design appropriate interventions to effectively interrupt the disease transmission (48) if new variants emerged.

## 5. Conclusion

This work highlights the effects of vaccination on the admission of COVID-19 patients in hospitals and ICUs in Germany. Our results showed a decrease in the number of patients in hospitals and ICUs for an increase in vaccine coverage. This is in line with the protective effects of vaccines against the severe forms of COVID-19 as proved through clinical trials. Moreover, we found that the response to vaccination could be delayed for about 20 days. These findings could be used for designing vaccination programs for future pandemics. Further studies should assess the effects of vaccination considering regional, demographic and social aspects.

## Data availability statement

Publicly available datasets were analyzed in this study. This data can be found here: https://www.corona-datenplattform.de.

## Ethics statement

Ethical review and approval was not required for the study on human participants in accordance with the local legislation and institutional requirements. Written informed consent from the participants' legal guardian/next of kin was not required to participate in this study in accordance with the national legislation and the institutional requirements.

## Author contributions

BEL, RGK, and MW contributed to the study conception and design. BEL carried out data analysis and wrote the initial manuscript draft. MW contributed to data collection, supervised data analysis, and revised the manuscript. RGK supervised data analysis and revised the manuscript. All authors read and revised the initial manuscript. All authors contributed to the article and approved the submitted version.
