# Supplementary material for: Lag-time effects of vaccination on SARS-CoV-2 dynamics in German hospitals and intensive-care units
Source: Front Public Health. 2023 Apr 11;11:1085991. doi: 10.3389/fpubh.2023.1085991 (PMC10126254; doi:10.3389/fpubh.2023.1085991)
Supplement: Supplementary file 1 [file Data_Sheet_1.PDF]

Supplementary Material

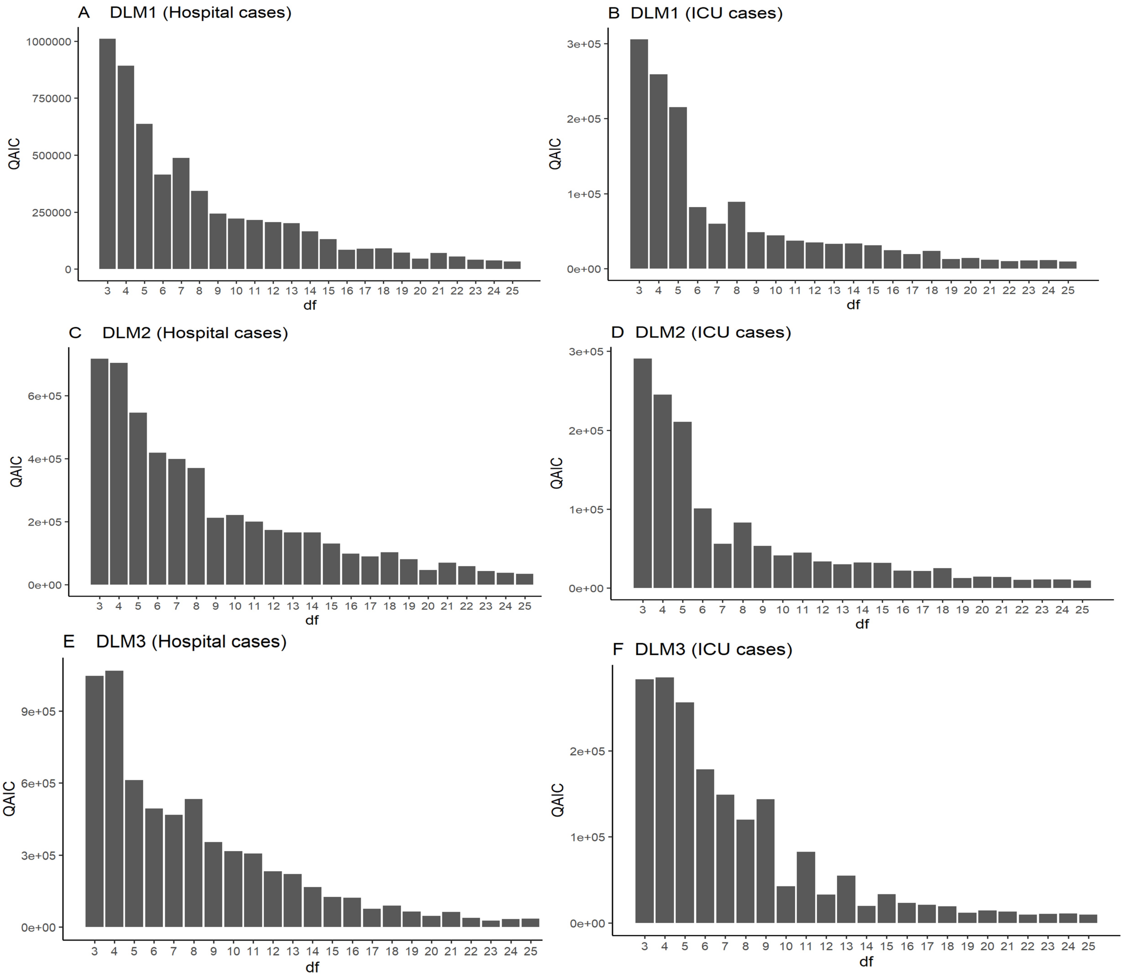

Figure S1. The quasi-Akaike Information Criterion (QAIC) values in function of the number of degrees of freedom (dfs) for adjusting for long-term trends of the variable time in the models. **(A)** QAIC and dfs for the model with the first dose V_1 and Hospital cases. **(B)** QAIC and dfs for the model with the first dose V_1 and ICU cases. **(C)** QAIC and dfs for the model with the second dose V_2 and Hospital cases. **(D)** QAIC and dfs for the model with the second dose V_2 and ICU cases. **(E)** QAIC and dfs for the model with the third dose V_3 and Hospital cases. **(F)** QAIC and dfs for the model with the third dose V_3 and ICU cases. DLM1, DLM2, and DLM3 represent the distributed lag models for the first, second and third doses.

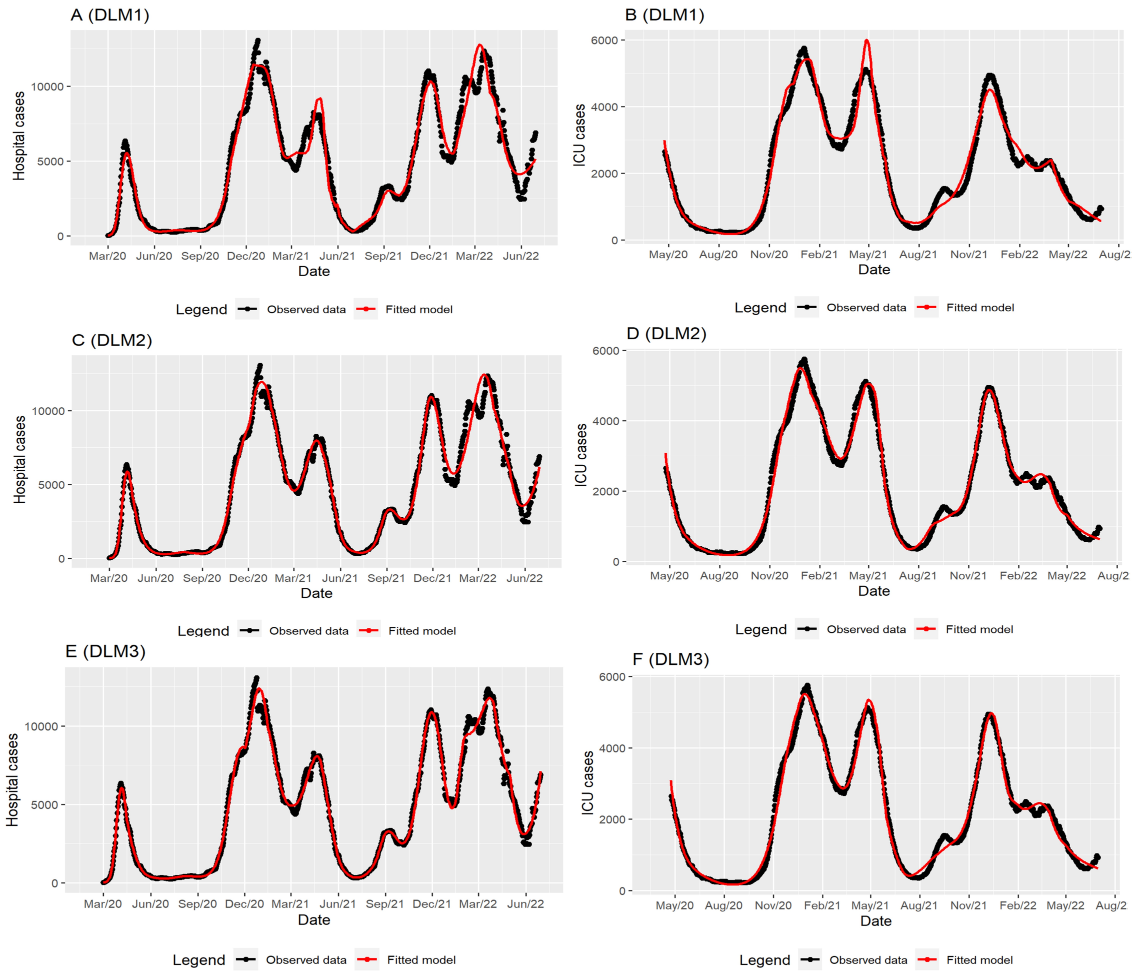

Figure S2. Models fitting with the optimal number of degrees of freedom. **(A)** Model fitted with df=16 (hospital cases). **(B)** Model fitted with df=15 (ICU cases). **(C)** Model fitted with df=20 (hospital cases). **(D)** Model fitted with df=16 (ICU cases). **(E)** Model fitted with df=23 (hospital cases). **(F)** Model fitted with df=16 (ICU cases).

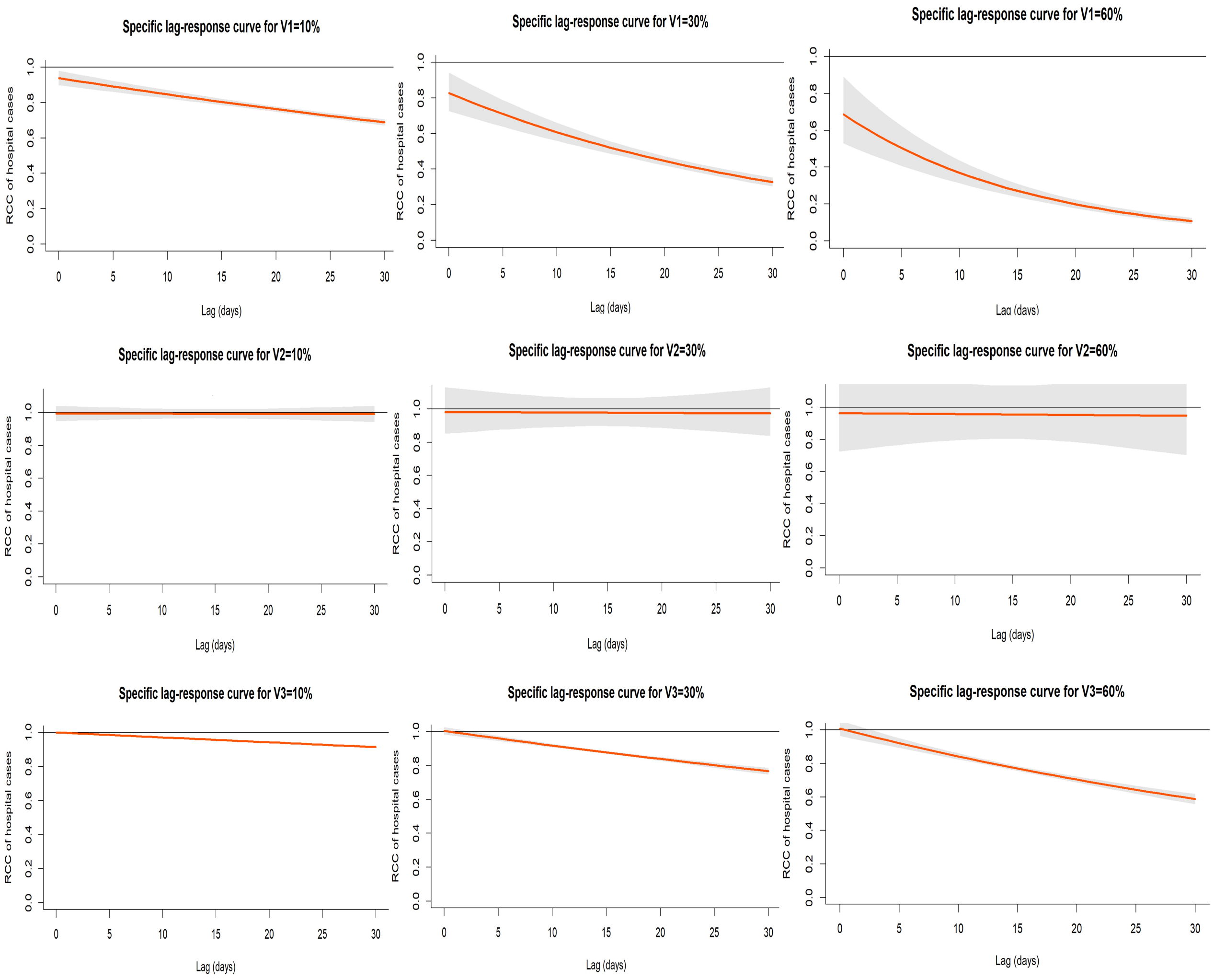

Figure S3. Specific lag-response curves showing RCC point estimates and their confidence intervals for vaccination coverages V1, V2 and V3 in the cases of hospital patients.

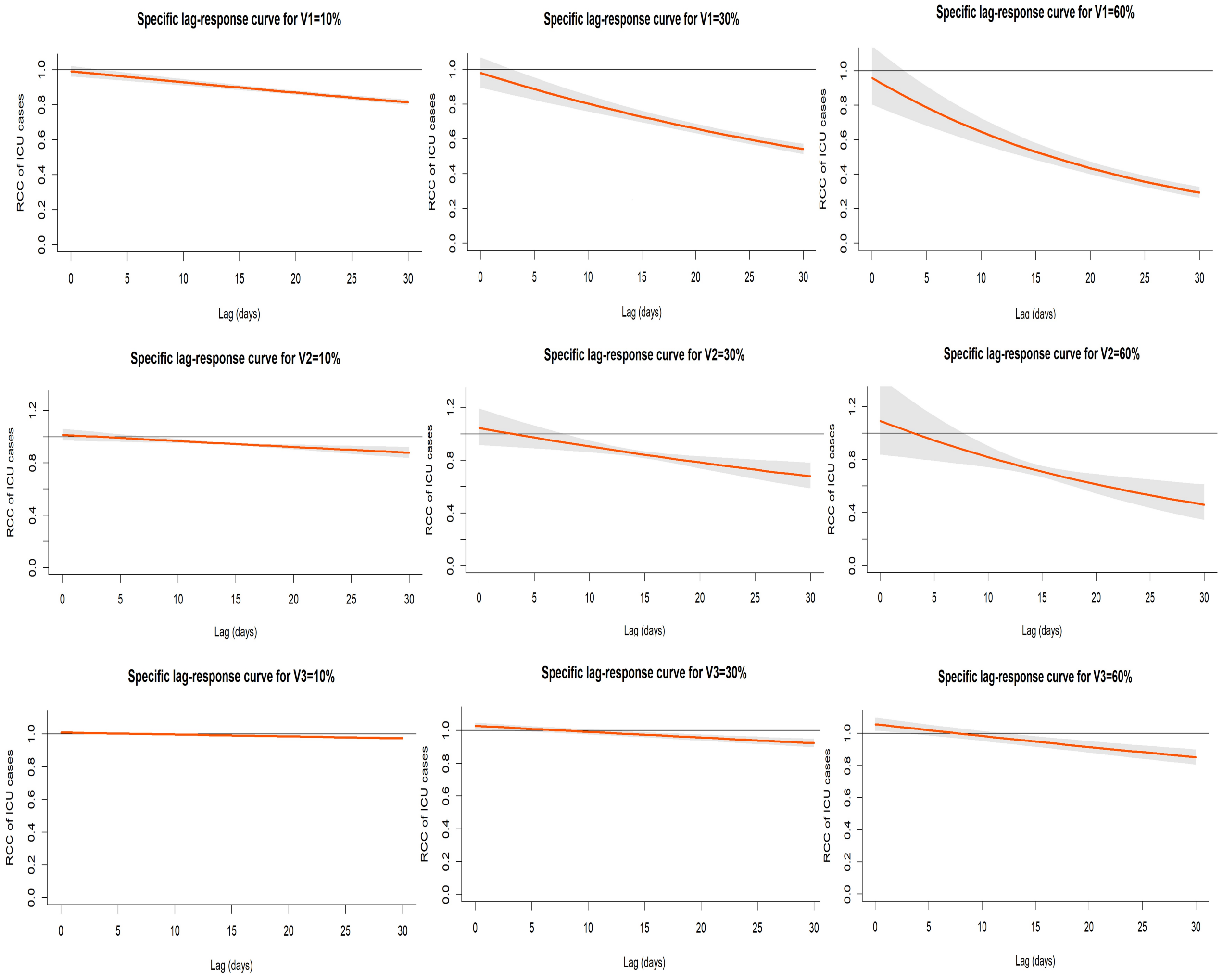

Figure S4. Specific lag-response curves showing RCC point estimates and their confidence intervals for vaccination coverages V1, V2 and V3 in the cases of ICU patients.
